# Future Perspectives of Prime Editing for the Treatment of Inherited Retinal Diseases

**DOI:** 10.3390/cells12030440

**Published:** 2023-01-29

**Authors:** Silja Hansen, Michelle E. McClements, Thomas J. Corydon, Robert E. MacLaren

**Affiliations:** 1 Department of Biomedicine, Aarhus University, 8000 Aarhus, Denmark; 2 Nuffield Department of Clinical Neuroscience, University of Oxford, Oxford OX3 9DU, UK; 3 Oxford Eye Hospital, Oxford Hospitals NHS Foundation Trust, Oxford OX3 9DU, UK; 4 Department of Ophthalmology, Aarhus University Hospital, 8000 Aarhus, Denmark

**Keywords:** CRISPR, prime editing, gene editing, inherited retinal diseases

## Abstract

Inherited retinal diseases (IRD) are a clinically and genetically heterogenous group of diseases and a leading cause of blindness in the working-age population. Even though gene augmentation therapies have shown promising results, they are only feasible to treat a small number of autosomal recessive IRDs, because the size of the gene is limited by the vector used. DNA editing however could potentially correct errors regardless of the overall size of the gene and might also be used to correct dominant mutations. Prime editing is a novel CRISPR/Cas9 based gene editing tool that enables precise correction of point mutations, insertions, and deletions without causing double strand DNA breaks. Due to its versatility and precision this technology may be a potential treatment option for virtually all genetic causes of IRD. Since its initial description, the prime editing technology has been further improved, resulting in higher efficacy and a larger target scope. Additionally, progress has been achieved concerning the size-related delivery issue of the prime editor components. This review aims to give an overview of these recent advancements and discusses prime editing as a potential treatment for IRDs.

## 1. Introduction

Inherited retinal diseases (IRD) are a group of hereditary disorders characterized by the combined dysfunction and degeneration of photoreceptor cells and/or the retinal pigment epithelium (RPE) leading to irreversible and progressive vision loss [1,2]. The genetic and clinical heterogeneity, with causative variants identified in >250 genes presenting highly distinct disease courses and phenotypes, constitutes a significant challenge for the treatment of these diseases [2,3]. IRDs affect approximately 1 in 3,000 people worldwide and are the leading cause of of vision loss in the working-age population, thereby providing an immense burden for society and the individual patient [4,5]. Hence, there is an urgent need for a development of therapies to cure IRDs. The eye´s unique features, including relative immune-privilege, compartmentalization, and visual and surgical access, appoint it an optimal target organ for gene therapy [6]. Encouragingly, gene augmentation therapy has shown to be successful for the treatment of autosomal recessive IRD and has experienced a major breakthrough with the FDA approval of voretigen-neparvovec in 2017 [7,8,9]. 

However, this treatment strategy is not feasible for (i) variants in genes that are too large to be encoded within the adeno-associated viral (AAV) vector, including *USH2A*, *ABCA4*, and *CEP290*, or (ii) autosomal dominant IRD, in which the disease-causing variant is gain-of-function or dominant negative and therefore requires allele specific silencing, which has not yet been optimized [2,10]. Thus, the majority of IRDs do not benefit from a simple gene augmentation approach and novel treatment strategies and are therefore the topic of interest for the pharmaceutical industry and clinicians and for the treatment of patients worldwide. 

Gene editing therapy is showing great potential to correct genetic disorders directly in the retina, and a phase I/II clinical trial for Edit-101 (AGN-151587), a treatment for Leber congenital amaurosis (LCA) type 10, is currently ongoing with no reported serious adverse reactions [11]. This recent advancement is based on the clustered regularly interspaced short palindromic repeats (CRISPR)/Cas9 technology that revolutionized the field of molecular medicine and gene editing. The discovery of CRISPR led to the award of the Nobel Prize in Chemistry to Jennifer Doudna and Emmanuele Charpentier in 2020 [12]. This technology is composed of two key elements: (i) the Cas9 endonuclease and (ii) a single guide RNA (sgRNA), that specifies the genomic target site. When engaged to its target sequence, the CRISPR/Cas9 complex induces a double strand break (DSB) and utilizes the endogenous DNA repair process through two major pathways: non-homologous end joining (NHEJ) or homology-directed repair (HDR) [13,14,15,16]. However, both strategies are problematic as the NHEJ repair pathway is error prone, resulting in frequent insertions/deletions (indels) while HDR requires a donor template and the mechanism is inefficient in non-dividing cells, such as the cells of the retina [10]. Additional pathways, including microhomology-mediated end joining (MMEJ) and the single strand annealing (SSA) pathway that both require 3´single strand DNA overhangs generated by DNA resection at the DSB site for the subsequent annealing of homologous sequences to realign the broken strands, lead to deletions and are more error prone [17,18]. Furthermore, double-strand breaks (DSBs) in the DNA induced by the Cas9 nucleases have been shown to cause off-target cleavage, large deletions, chromosomal rearrangements, chromothripsis, and activation of p53 [19,20,21,22,23,24,25].

**Figure 1:**
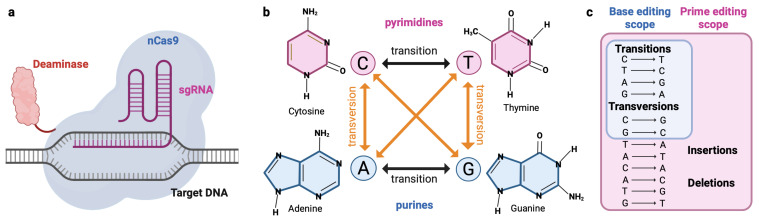
(**a**) The base editing system consists of a single guide RNA (sgRNA), a Cas9 Nickase (nCas9) and a deaminase enzyme (adenin deaminase for ABEs and cytosine deaminase for CBEs). (**b**) Graphical overview of the 12 different base pair substitutions. (**c**) Editing scope of the base editing (blue) and prime editing (pink) technology.

These risks and limitations of DSB-based CRISPR/Cas9 gene editing have led to the motivation to develop DSB-independent CRISPR/Cas systems such as base editing and prime editing. Base editors (BEs) consist of three key components: (i) a sgRNA, (ii) the Cas9 nickase (nCas9) for programmable DNA binding and introduction of a single-stranded DNA nick, and (iii) a deaminase enzyme that enables targeted base conversion [26,27,28]. Collectively, cytosine base-editors (CBEs), and adenine base-editors (ABEs), enable the correction of all four transition mutations (C>T, T>C, A>G and G>A). Recently, researchers have expanded the target scope of BE to the correction of two transversion point-mutations [29]. Compared to gene editing with Cas9 nucleases, base editing showed superior editing efficacies and less DSB-related undesired outcomes (Figure 1) [30].

However, base editing systems also have limitations including: undesired “bystander editing“ when multiple cytidines or adenines are present in the targeted region, and base editing window restriction due to the requirement of a protospacer adjacent motif (PAM) within 15 +/- 2 nt from the targeted nucleotide [31]. Additionally, some BEs have shown to induce off-target mutations in both DNA and RNA [32,33]. Lastly, the target scope of base editors is restricted to the correction of six of the 12 types of point mutations and excludes insertions and deletions. As transversion mutations account for about 20% of the >75.000 known disease-associated human genetic variants, insertions and deletions account for approximately 30% of known pathogenic human variants [34], which make a more versatile gene editing tool desirable.

**Figure 2:**
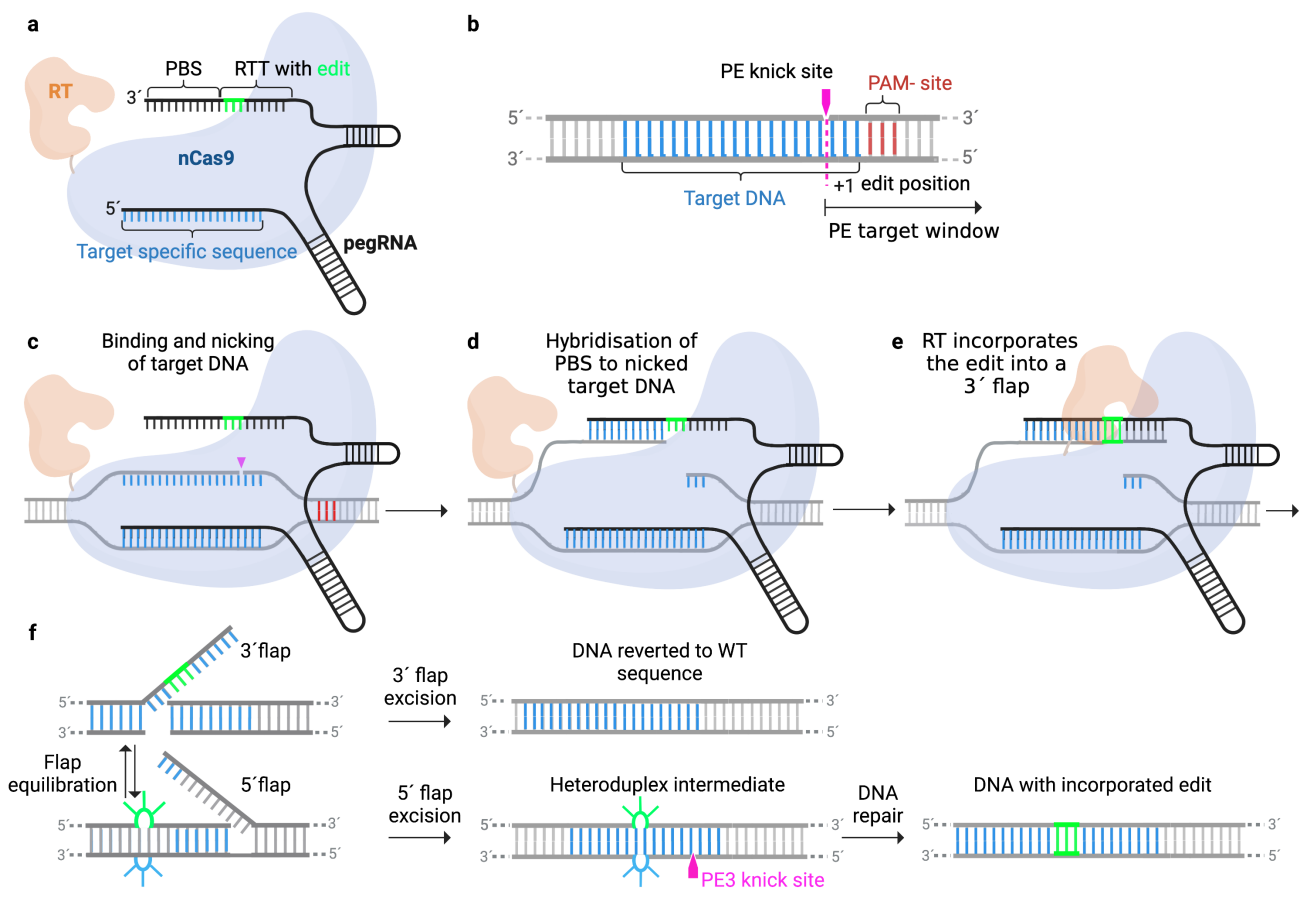
Graphical overview of the prime-editing technology. (**a**) The prime-editor complex consists of a H840A SpCas9 nickase (nCas9) fused to a reverse transcriptase (RT) and a prime editing guide RNA (pegRNA) containing the primer binding site (PBS) and the RT template (RTT) encoding for the desired edit (green). (**b**) Detailed presentation of the target DNA. (**c**) The PE complex binds to the target DNA and nicks the PAM-strand. (**d**) The PBS anneals to the nicked target DNA and (**e**) enables the RT to start reversed transcription using the RTT as a template. This induces a 3´flap with the desired edit. (**f**) The 3´flap equilibrates with the unedited 5´flap. Excision of the 5´flap by endonuclease produces a heteroduplex intermediate consisting of one DNA strand with the desired edit and the complementary strand without the edit. DNA repair of the unedited strand results in permanent incorporation of the desired edit on both DNA strands. Nicking of the unedited strand with a conventional sgRNA biases repair towards installation of the edit.

Prime-editing is the newest addition to the CRISPR-Cas9 toolbox, first described by Anzalone et al. in 2019 [35]. Prime editing does not induce a DSB and can be used to precisely correct all types of point mutations, insertions, and deletions, thereby having the potential to repair approximately 89% of all human pathogenic genetic variants [35]. The prime editing technology requires two key components: (i) the prime editor (PE), consisting of a reverse transcriptase (RT) fused to a H840A SpCas9 nickase (nCas9) and (ii) the prime editing guide RNA (pegRNA), functioning both as a guide to the target site and as a template for the desired edit. The pegRNA is comprised of the primer-binding site (PBS) which serves as a docking site for the RT and the RT-Template (RTT) which includes the desired edit. The mechanism for prime editing is illustrated in (Figure 2). 

Briefly, the PE/pegRNA complex binds to the target DNA and the nCas9 nicks the PAM strand. Then, the primer binding site of the pegRNA hybridizes to the resulting 3´flap, and the RT initiates the extension of the PAM strand and incorporates the desired edit using the RTT as a template. This process creates two competing intermediate flaps: the 3´flap with the intended edit and 5´flap with the unintended original sequence. Thermodynamically, the unedited, perfectly complementary 5´flap is likely to be favored for incorporation. However, during lagging-strand DNA synthesis occurring endonucleases, such as FEN1 favor 5´flaps as their substrate, which shifts the balance towards degradation of the 5´flap and incorporation of the 3´flap [35,36]. Finally, the resulting heteroduplex DNA strand is resolved and the desired edit is integrated into both strands of the target DNA by endogenous DNA repair mechanisms. Prime editing has shown to be more effective than HDR [35] and compared to base editing, prime editing has a larger target scope and may be superior for targets with the risk of bystander edits [35].

Since its initial discovery in 2019, several publications have shown successful application of prime editing in several cell lines, postmitotic neurons [35], mice [37], organoids, [38] and plants [39,40,41]. However, the prime editing technology is still in its infancy and a number of obstacles have yet to be overcome to make it approachable as an effective and broadly applicable gene editing tool. In this review, we describe the development and optimization of the prime editing technology, as well as current challenges and limitations. Furthermore, we discuss prime editing as a future approach for the treatment of inherited retinal disease.

## 2. Development and Improvement of the Prime Editing Technology

In the seminal publication, Anzalone et al. described 3 different versions of the prime editing system: PE1, PE2, PE3. The first generation of prime editors (PE1), consist of a wild-type Moloney murine leukemia virus RT (M-MLRV RT), fused to a nCas9 with a separate plasmid encoding the pegRNA [35]. This system successfully introduced transversion point mutations with an editing efficacy of 0.7–5.5% and installed insertion and deletions with 4–17%. For PE2, five mutations (D200N/LL603W/T330P/T306K/W313F) were introduced into the M-MLRV to increase thermostability, binding, and enzyme processivity. These changes yielded a 1.6–5.1-fold improvement of editing efficacy. To further enhance prime editing efficacy, a single guide RNA (sgRNA) was designed to induce a distal nick on the non-edited strand. Thereby, DNA repair is directing to that strand and the edited strand is utilized as a template. This version, termed PE3, further improved editing efficacy 1.5 to 4.2-fold compared to PE2 with editing rates of up to 55% [35]. However, PE3 was associated with increased indel formation, possibly due to the nicking of both strands at roughly the same time. To circumvent this issue, sgRNA with a spacer that only matched the edited strand but not the original sequence, were designed. This refined approach was named PE3b and led to similar editing outcomes but with a 13-fold decreased indel formation compared to PE3. Moreover, in cases where the RT-templates could be designed to silence the PAM site, increased editing specificity was observed, possibly due to the inability of the PE complex to re-bind and re-nick the target DNA. The authors recommend using this approach whenever possible [35]. 

Since this first publication, several researchers have engineered and modified the prime editing components with the aim of increasing editing efficacy, broadening the scope of target mutations, and to enhancing the understanding of the prime editing method. For this, various strategies have been applied. 

### 2.1. Engineering the Prime Editor Protein

Liu et al. hypothesized that a nuclear localization signal (NLS) sequence-optimized prime editor may increase prime editing efficacy, especially in post-mitotic or quiescent cells, where the nuclear barrier may be a greater hurdle for the PE components [42,43]. Therefore, the authors developed the PE* system, comprising of a N-terminal c-Myc NLS, and both a variant bipartite SV40NLS (vPB-SV40) and SV40 NLS at the C-terminus. The PE* method outperformed conventional PE2 when applied to established base-pair-substitutions, insertions and deletions in reporter cell-lines (1.3 to 2.1-fold), at different endogenous loci (1.4 to 3.4-fold) and in vivo (3.1-fold). However, when applied with an additional nicking sgRNA the frequency of undesired indels was equally or slightly higher compared to conventional PE3. Furthermore, PE* was successfully delivered to mouse liver using a dual AAV strategy split-intein approach, enabling the correction of a pathogenic E342K mutation in SERPINA1 with a editing efficacy of 3.1 +/- 0.6% [43]. 

To stabilize the single stranded DNA (ssDNA) that is generated when the pegRNA binds to its target sequence and is used as a primer for reverse transcription, Song et al. included a Rad51 ssDNA-binding protein between nCas9 and the RT. This variant was named hyPE2 and yielded a 1.4 to 1.5-fold higher editing efficacy for different endogenous target sites. The authors speculated the Rad51 mediated binding of the pegRNA to the nicked target ssDNA increased reverse transcription, thereby leading to the increased editing efficacy observed in this study [44]. A similar approach has previously shown to increase base editing efficacy [45]. 

Velimirovic et al. utilized a high-throughput approach based on a library of 12,000 amino acid peptides fused to the N-terminus of PE2 to investigate the influences of peptide fusion on prime editing efficacy [46]. Based on the best performing dual-peptide-PE2 fusion, the IGFpm1-NFATC2IPp1-PE2 (IN-PE2) was established as an improved prime editor platform. When employed to edit various targets in human and mouse cell lines, IN-PE showed improved editing efficacies compared to the conventional PE2 system. The authors postulated that the observed increase of efficacy may be due to increased translation efficacy that enhances the level of PE2, but stated that the mechanisms behind this process remain an open question.

Zong et al. modified the M-MLV RT by removing its ribonuclease H domain and by incorporating a viral nucleocapsid protein with nucleic acid chaperone activity [47]. This engineered plant prime editor (ePPE) increased editing efficacy 5.8-fold compared to the conventional PPE system [39] for substitutions, deletions and insertions in plant cells, without leading to formation of undesired byproducts. Unfortunately, this strategy did not result in higher editing efficacy in human cell-lines.

### 2.2. Improving pegRNA Stability and Structure

Different studies have shown that the binding stability of 3´end of pegRNA to the target DNA is a key factor for PE efficacy, as it is essential for the process of reverse transcription [48]. Kim et al. described that high GC-content and GC-count of the PBS, leading to a stable binding of pegRNA to target DNA, is one of the most important features for prime editing efficacy and influences pegRNA design for different target sites [48]. 

Nelson et al. hypothesized that degradation of the 3´end of pegRNA resulting in loss of the PBS may impede PE editing efficacy by occupying the PE and target site for functional pegRNAs. Therefore, the authors extended the 3´ termini of pegRNAs with structured RNA motifs (evopreQ_1_ or mpknot), which increased stability and prevented degradation of the 3´end. The engineered pegRNAs (epegRNAs) improved PE efficacy in several cell types (HeLa, U2OS, K562 and human fibroblasts) approximately 3 to 4-fold compared to conventional PE3, without increasing indels rates. Furthermore, a computational platform (pegLIT) to identify non-interfering 8bp nucleotide linkers between the RNA-motif and epegRNA was developed [49]. 

Similarly, Zhang et al. incorporated a viral derived exoribonuclease-resistant RNA motif (xrRNA) with the aim to prevent 3´terminus degradation [50]. This novel platform was named xrPE and enhanced editing efficacy in different cell-lines up to 2.5, 3.1 and 4.5-fold compared to conventional PE3, while the ratio of desired edits to undesired indels (edit:indel ratio) were similar. A direct comparison to the epegRNA platform showed equivalent editing performances.

Using a related strategy, Li et al. extended the 3´terminus of pegRNA with a human telomerase RNA (hTR) G-quadruplex modification. This platform was named G-PE and yielded similar editing outcomes compared to prime editing with epegRNAs an Xr-PE and has the advantage of a smaller size [51].

Li et al. described a strategy to stabilize the secondary structure of pegRNA itself: the backbone of the pegRNA was amended by changing each non-G/C pair to a GC pair in the small hairpin of pegRNA [52]. This method was termed aPE and resulted in editing efficacies which were on average 2.77-fold improved compared to a regular PE3 system. Additionally, the same authors developed the aPE strategy, where small same-sense mutations (SSMs) were introduced in the RTT of pegRNA. Design rules for this SSM were established stating that SSMs at position 1, 5, 6, 2/5, and 3/6 were most favorable and led to PE efficacies for single bases with an average 353-fold increase compared to conventional PE3. A combination of the aPE and sPE could further improve PE efficacy and integration in the PE5 system (sPE5) outperformed the classical PE5 strategy.

Liu et al. hypothesized that prime editing efficacy may be impeded by the circularization of pegRNA due to complementarity of spacer and 3´end [53]. To solve this problem, the authors designed pegRNA with a 20-nt Csy4 recognition site at the 3´end, which prevented circulation by formation of a hairpin structure and fused Csy4-T2A to Cas9 Nickase. This enhanced the prime editing method (ePE) and resulted in increased editing efficacy but unfortunately also higher indels.

Several researchers have replaced the original pegRNA scaffold with the `flip and extension (F+E) sgRNA scaffold, that has previously shown to increase Cas9 activity. This modification extends one of the scaffold hairpins, thereby disrupting a a Pol III terminator sequence, and has shown to increase prime editing efficacy for some target sites [49,54].

### 2.3. Suppressing DNA Repair Mechanisms

Chen et al. explored the cellular determinants involved in DNA repair mechanisms with the aim to identify elements that may improve prime editing efficacy. Utilizing a pooled CRISPR interference-based screen the authors identified specific DNA mismatch repair (MMR) genes that substantially impede prime editing efficacy and increase the formation of undesired indels [55]. Hence, the authors speculated that DNA mismatch repair (MMR) hampers PE efficacy by promoting excision of the intermediate 3´flap with the desired edit thereby favoring integration of the unedited 5´flap. Furthermore, they discovered that siRNA-based knockdown of MMR in HEK293T cells led to increased prime editing efficacy (from 7.7% to 25%) and reduced indel frequency (from 0.39% to 0.28%). This is in alignment with a recent study publish by Da Silva et al. [56], which showed that suppression of mismatch repair (MMR) increased prime editing efficacy 2–17-fold in different cell lines for various types of edits. 

Encouraged by these results, Chen et al. developed a novel prime editing platform which is based on the co-delivery of prime-editor and the engineered MMR-inhibiting protein MLH1dn [55]. This system was named PE4 (when combined with PE2) or PE5 (when combined with PE3) and enhanced the installation of base substitutions, small insertion and deletion by an average of 7.7-fold and 2-fold compared to the PE2 and PE3 system, respectively. Additionally, the authors demonstrated that designing pegRNAs to introduce silent mutations near the target site, can cause the PE intermediate to evade MMR. Moreover, this study improved the PE2 architecture by including a human codon optimized reverse transcriptase, a 34-aa linker containing a bipartite SV40NLS [57], an additional C-terminal c-Myc NLS [58], and R221K, N394K mutations of SpCas9 [59]. This new system was named PE2max, PE3max, and PE5max, respectively, and outperformed the conventional PE2 architecture by 2.8-fold in MMR proficient HeLa cells and 1.2-fold in in MMR deficient HEK239T cells. Lastly, the authors combined the PEmax system with epegRNAs showing 72-fold enhanced editing efficacy for PE4max in MMR proficient cells and 12-fold-enhancement for PE5max in MMR-proficient cells and reduced indel outcomes, compared to conventional PE2 and PE3, respectively. In conclusion, the use of PE5max was recommended for most efficient PE [55].

### 2.4. Improving Accessibility of the Target DNA

Park et al. hypothesized that opening the chromatin structure of the DNA target site may increase prime editing efficacy. To achieve this, two strategies were developed: firstly, inspired by the proxy-CRISPR strategy [60] the authors designed proximal dead sgRNAs (dsgRNA), a 14- or 15-nt guide RNA [61] with the ability to unwind the chromatin structure of the target site. Thus, the prime editor was utilized to both induce prime editing with a pegRNA and to modulate the neighboring chromatin structure with a dsgRNA. Secondly, the PE was engineered using the chromatin-modulating peptides (CMPs) high mobility group nucleosome binding domain 1 (HN1) and histone H1 central globular domain (H1G). Collectively, this enhanced prime editing, achieving 2.55–5.11 -fold higher editing efficacy compared to PE3 when applied to edit different target sites in mouse cell lines [62]. Furthermore, this method was used to generate *lgf2* mutant mice with editing frequencies up to 47%. These results are in alignment with a study published by Yao and colleagues, identifying a group of histone deacetylase inhibitors (HDACi), that increased efficacy of PE meditated insertions and deletions by promoting an open chromatin state of the target DNA [63]. 

Collectively, these observations support the assumption that opening the chromatin structure may increase prime editing efficacy by increasing accessibility of the target DNA for the PE/pegRNA complex to bind.

### 2.5. Utilizing Two pegRNAs

To improve prime editing efficacy further and/or to facilitate deletion or insertion of larger DNA fragments, several studies have established approaches utilizing a pair of guide pegRNAs. 

Anzalone et al. developed the TwinPE strategy, which consists of two pegRNA targeting opposite strands and synthesizing new 3´flaps that are complementary to each other. Thereby, TwinPE enables direct introduction of the desired edit on both strands instead of requiring the cell to synthesize the other strand that was not edited. This strategy could successfully replace, insert or delete DNA larger sequences in various human cells. Furthermore, in combination with a site-specific serine recombinase, integration of gene-sized DNA plasmids (>5000 bp) and target sequence inversions of 40 kB in human cells was achieved [64]. Similarly, Wang and co-workers developed GRAND editing (genome editing by RTTs partial aligned to each other but non-homologous to target sequences within duo pegRNA), to precisely insert large DNA fragments ranging from 20bp to 1 kb reaching editing efficacies of up to 63% for a 150 bp insertions and 28.4% for a 250 bp insertion [65].

To enable installation of precise point mutations, small insertions and deletions, Zhuang et al. developed a similar approach, named homologous 3´extension mediated prime editor (HOPE). HOPE showed higher editing efficiency and fewer undesired indels compared to PE3 when tested in HEK239T cells and human colorectal carcinoma 116 (HCT116) cells [66].

Furthermore, Choi et al. established the PRIME-Del platform, to use prime editing for efficient deletion of larger DNA sequences. In contrast to TwinPE and GRAND, the pegRNA encodes for 3´flaps that are not only complementary to each other, but also to the DNA sequence upstream of the nick on the opposite DNA strand. The authors used this method to introduce deletions up to 10 kb with 1–30% editing efficacy and to combine genomic deletions with small insertions. Compared to the Cas9n/paired sgRNA strategy, PRIME-Del demonstrated higher precision in various genomic loci [67]. Similar to PRIME-Del, Tao et al. published the bi-directional prime editing platform (Bi-PE) [68], that achieved increased editing efficacy compared to the PE3 system when utilized to install conversion of multiple bases.

Jiang et al. developed the PE-Cas9-based deletion and repair (PEDAR) strategy, which is comprised of an active Cas9 nuclease instead of a nickase. PEDAR was utilized to introduce >10 kb deletions and up to 60 bp insertions in cells and could successfully remove a 1.38 kb large pathogenic insertion of the Fah gene in a mouse model of tyrosinemia and restore Fah expression in the liver [69]. However, compared to PRIME-Del, this method was more error prone.

### 2.6. Additional Strategies

Recently, Abudayyeh and colleagues described the Programmable Addition via Site-specific Targeting Elements (PASTE) platform. This technology consists of a CRISPR-Cas9 nickase fused to a reverse transcriptase and an additional serine integrase which enables the integration of sequences with a size of up to 36kb in different human cell line and primary T cells and primary human hepatocytes. Similar or improved editing efficacy compared to HDR and non-homologous end joining based integration was observed and fewer off-target events [70]. Furthermore, Adenovirus (AdV) based delivery to liver-humanized FRG mice in vivo via retro-orbital injection resulted in integration rates of up to 2.5%.

**Figure 3:**
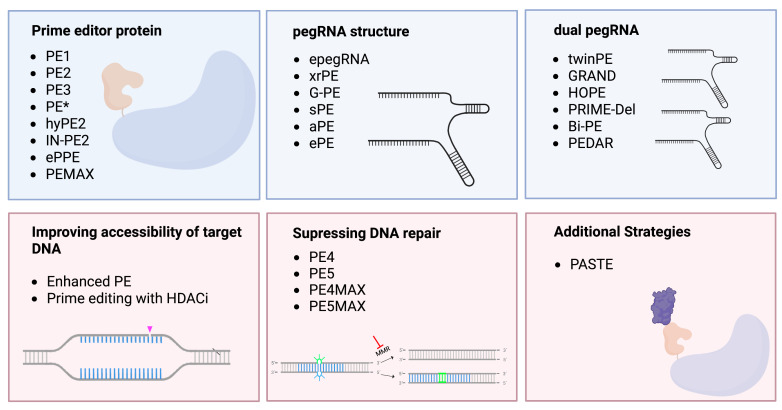
Graphical overview of the development and improvements of the Prime editing technology. Improvements affecting the PE:pegRNA complex in the upper row (blue), other improvements in lower row (pink).

Collectively, the efforts described above and summarized in Table 1 and Figure 3, have significantly increased both efficacy and target scope of the prime editing technology since its first description in 2019. As these improvements address different steps in the prime editing mechanisms, i.e., translation of the PE protein, transport of the PE to the cell nucleus, accessibility of the target site, stability of the PE/pegRNA/target-site complex and incorporation of the edited strand, its combination may further improve PE efficacy. With many options currently available, selection of the appropriate PE construct for a given experiment depends on (i) the desired edit and (ii) the target cell type. When targeting point mutations, small insertions or deletions, the PE3 system with the PEmax protein architecture (PE3 max system) combined with an epegRNA may be a good choice, as this combination addresses several aspects to improve prime editing efficacy and has previously shown to increase efficacy significantly [55]. For MMR-competent cells, co-delivery of MLH1dn, to inhibit MMR may further improve the editing efficacy (PE5max system). Additionally, utilizing PE3b/PE5b when possible may decrease formation of undesired indels. However, in cases where the additional nicking sgRNA causes an unacceptable amount of indel formation, the PE2max system (PE2 + PEmax architecture) or PE4max system (PE2 + PEmax architecture + MLH1dn) may be preferred over PE3max and PE5max [71]. Plasmids for this approach are available on Addgene: epegRNA cloning vector (pU6-tevopreq1-GG-acceptor, Addgene ID: 174038), PEmax Plasmid (pCMV-PEmax, Addgene ID: 174820), hMLH1dn (pEF1a-MLH1dn, Addgene ID: 174824) and sgRNA cloning vector (pU6-pegRNA-GGacceptor, Addgene ID: 132777). Furthermore, for detailed guidelines for selection of PE system and pegRNA and protocol for executing prime editing experiments in mammalian cells, the recently protocol published by Doman et al. is recommended [71].

When targeting larger deletions or insertions of 100-200bp, dual pegRNA approaches as, i.e., TwinPE or GRAND may be favorable. Lastly, when aiming for integration or inversion of very large sequences >5000 bp strategies including a serine integrase, like PASTE or TwinPE (in combination with Bxb1 recombinase) are the option of choice. Additionally, to advance the efficacy of the prime editing system, these studies have led to an increased understanding of the prime editing technology, which may be utilized to further refine this technology in the future.

## 3. Identification of Successful pegRNA Candidates

Currently, identification and validation of efficient and target specific pegRNA candidates provide a huge challenge for researchers and often require labor-intensive experiments and limited success. To optimize this process, a careful pegRNA design and an appropriate screening approach is essential. 

### 3.1. Design of pegRNAs

Previous studies have shown that a thorough design of the pegRNA is essential for high prime editing efficacy and specificity [35,48]. This includes: (i) the length and G/C-content of PBS, (ii) the length of RTT, (iii) the position of the RT initiation site in relation to the target site, (iv) the composition of the desired sequence alteration, and (iii) the position of the alternate strand nick (for PE3) [35,43]. Based on prime editing results in various cell lines the original publication suggests the following parameters as starting point for pegRNA design: (i) a PBS length of 13 nt, which should be increased in case of low G/C content, (ii) a RTT length of 10-16nt, (iii) an 3´extension that does not start with C, and (iv) a 50bp distance relative to the initial nick site for the alternated strand nick [35]. 

In a high throughput analysis based on a paired library approach, Kim et al. identified the DeepSpCas9 score, G/C counts in PBS, and Tm of PBS as key factors for prime editing efficacy. Hence, the authors extended the initial design guidelines by the following recommendations (i) PBS extension to 15 nt when the GC content is <40% and abbreviation to 9 nt when the GC is >60% (ii) alteration of the RTT to 11–15 nt in favor to end with a G nucleotide (iii) PAM editing whenever possible [48]. 

Recently, Doman et al. published a protocol for initial design of pegRNA candidates for experiments in mammalian cells, including recommendations for the design of epegRNAs and TwinPE pegRNAs [71]. 

Based on some of these recommendations, several in silico design tools (Table 2) have been developed to automatically design the most efficient pegRNA and nicking sgRNA (PE3) candidates to introduce or repair a desired mutation [72,73,74,75,76,77,78].

To specify the desired target locus, the in silico tools require either manual input of the reference sequence and the desired sequence of the DNA target site [73,74,77] or include the possibility to define the target site from a reference genome [72,77] or the ClinVar database [76,78]. Depending on the tool, pegRNA design can be customized for different Cas9 orthologues [73,74,76,78] and design parameters as PBS and RTT length, editing strategy (PE2, PE3, PE3b), PAM silencing [75,78], or sgRNA nicking distance [73] can be manually chosen. The provided candidates for pegRNA spacer, PBS, RTT, and nicking sgRNA are ranked according to previously described guidelines [35,48] and, for some tools, according to on-target and/or off-target scores [76,78]. Some of the tools provide a graphical overview of pegRNA/nicking sgRNA location on the target sequence [74,78] or visual display of the expected pegRNA secondary structure [75]. To prove applicability in vitro, the developers have shown successful prime editing in human cells line using in silico designed pegRNAs [73,75].

Recently, different design tools based on machine learning have been developed with the focus on predicting prime efficacy of pegRNA candidates. DeepPE is a webtool that provides prime editing efficacy for a total of 57 pegRNA per entered target sequence, varying in PBS and RTT length and introducing various edits [74]. Likewise, Li et al. developed Easy-Prime, which was trained using several previously published prime editing datasets. Easy-Prime utilizes the contribution of established and newly discovered features, as predicted RNA folding and secondary structure to prioritize pegRNA candidates and predict their editing efficacy. Additionally, this software enables the design of epegRNAs and provides optimized pegRNA design for 89.5% of 152351 GWAS variants [79].

**Table 2:** In silico tools for pegRNA design.

Name	Summary of Key Features	ON/Off Target Sore	Web Access	Ref
**PINECONE** (Prime Induced Nucleotide Engineering Creator of New Edits)	Provides reference genome of different organisms for definition of target sequence Provides cloning nucleotides, PCR, and sequencing primers for target locus	provides MIT score (UCSC browser) for pegRNAs specificity, candidates with highest score are chosen	https://github.com/xiaowanglab/PINE-CONE	[72]
pegFinder	Enables pegRNA design for different Cas9 orthologues (Cas9-NGG, Cas9-NG, and Cas9-SpRY)	Provides Optional sgRNA on/off-target score (based on Broad sgRNA designer [80,81] and CRISPscan [82])	http://pegfinder.sidichenlab.org	[73]
PE-Designer	Enables pegRNA design for different Cas9 orthologues (SpCas9, SpCas9-VQR, SpCas9-SpRY, SpCas9-VRER, NG-Cas9, iSpyMac Cas9, and xCas9) Supports pegRNA design for 543 different organisms Provides visual interface displaying location of pegRNA and nicking sgRNA on target DNA	Provides evaluation of potential off-targets for 544 different organisms (based on Cas-OFFinder [83])	http://www.rgenome.net/pe-designer/	[74]
PrimeDesign	Supports pegRNA design for single and combination editing applications and genome-wide and saturation mutagenesis screens Provides visually display of pegRNA secondary structure Includes PrimeVar database with candidate pegRNA and nicking sgRNA combinations for > 68.500 pathogenic human genetic ClinVar variants	Provides CFD score for spacer specificity [80]	https://primedesign.pinellolab.partners.org	[75]
Prime Editing design Tool	Provides pegRNA design for ClinVar Variants (correction or introduction) Custom Target sequence can be defined with ClinVar variant ID or Gene ID (no need for manual entry of target sequence) Supports pegRNA design for different Cas orthologues (Cas9-NG, xCas9, xCas9-NG or Cas9SpRy),	Includes pegRNA with Hsu-Scott off-target score [84] >50 only	https://primeedit.nygenome.org	[76]
PnB designer	Provides pegRNA design for single or multiple targets for different organisms Accepts sequence input or genomic coordinates as input format for target sequence Additionally enables design of Base editing guide RNAs		https://fgcz-shiny.uzh.ch/PnBDesigner/	[77]
pegIT	Provides design of single and multiple pegRNAs Accepts gene name or gene ID (reference genome for different organisms is available), Clinvar ID, or manual input of WT and edited target sequence as input format for target sequence Supports pegRNA design for different Cas9 orthologues (spCas9, CjCas9, SaCas9, SaCas9KKH, SpCas9NG) Modular structure allows rapid adjustment for new prime editing variants and CRISPR scoring rules	pegRNAs are ranked by ON-target score (based on CFD score [80]), Provides off-target evolution (including 0–3 mismatches)	https://pegit.giehmlab.dk	[78]
Easy-Prime	Machine learning based design tool Provides predicted editing efficacy for top pegRNAs and nicking sgRNA combinations Includes prediction of RNA folding in design of pegRNA candidates Provides visual interface displaying location of pegRNA and nicking sgRNA on target DNA v1.2 includes Option for EpegRNA design Provides pegRNA design for 89.5% of 152.352 GWAS variants	Provides Spacer specificity (based on DeepSpCas9-Score)	http://easy-prime.cc	[79]
DeepPE	Machine learning based Provides expected prime editing efficacy for 57 pegRNA candidates per target sequence Provides pegRNA candidates to generate different types of edits (deletions, insertions, substitutions)	provides Spacer specificity (based on DeepSpCas9 Score [85])	http://deepcrispr.info/DeepPE	[48]

### 3.2. pegRNA Screening Approaches

Following in silico design (Table 2), appropriate in vitro screening is then required. Doman et al. recommend to screen pegRNA candidates in easy-to-transfect workhorse cells like HEK293T cells or murine N2A cells. However, when targeting disease-specific mutations such as those that cause inherited retinal disease, multiple screening assays may be required. These would vary from reporter assay pegRNA screening to cell models carrying the specific pathologic variant. Hence, Anzalone et al. firstly employed prime editing to install a transversion mutation in HEK293T cells in the hemoglobin subunit beta (HBB) gene, which causes sickle cell disease. HEK239T cells homozygous for the mutated *HBB* allele were then isolated and used to screen pegRNAs for correction to wild-type HBB [35]. Although feasible for any mutation of interest, this process is labor intensive and requires the design of several pegRNAs to introduce and revert the mutation.

Using patient derived cells with the mutation of interest is another option to be considered [38]. However, these cells may be difficult to obtain and can be challenging to transfect. While patient derived cells are a well-suited model in terms of clinical translation, it may not be ideal for the initial high throughput screening of several pegRNA candidates.

Schene et al. developed fluoPEER, a fluorescence-activated cells sorting (FACS) based reporter system that can be amended to any genomic target site and enables high throughput screening of pegRNAs and Prime editor variants [86]. This reporter system is based on the fluoPEER plasmid, in which the genomic target sequence is inserted between an eGFP and an mCherry cassette. If the genomic target mutation is a frame-shift or nonsense mutation, the reporter assay will express eGFP and successful correction with prime editing will enable the expression of mCherry will be expressed. Editing efficacy of pegRNA variants and prime editor candidates are than ranked based on the ratio of mCherry to eGFP signal. Screening results with fluoPEER have shown a high correlation with results obtained with genomic editing, proving this system to be a reliable screening assay for both pegRNA and prime editor candidates. Similarly, Simon et al. established PEAR. In this reporter system, successful prime-editing activates a splice site leading to GFP expression [87].

Reporter based screening assays have the advantage of being easily amended to the desired target mutation and are ideal to for high throughput screens of both pegRNA and prime editor candidates. However, applying prime editing in vivo involves other important features not accounted for in reporter assays such as secondary structure and DNA target accessibility. Thus, top candidates from the pegRNA screening may perform less efficiently when applied on an endogenous target.

Another approach to identify the most successful pegRNA candidates for a given target, may be to utilize the high throughput approach described by Kim et al. However, this approach is based on a lentiviral library containing the target sequence rather than an endogenous target and thereby does not account for factors as secondary structure and DNA target accessibility [48].

## 4. Delivery of Prime Editing Components In Vivo for Treatment of IRD

In previous in vitro studies, plasmid-based lipid transfection has been used to deliver PE components to various mammalian cell lines [35]. Furthermore, electroporation has been utilized for plasmid-based delivery in patient-derived cells and organoids [38,88] and for delivery of in vitro-transcribed PE mRNA together with chemically modified synthetic epegRNA in patient-derived iPSCs, primary human T cells and patient-derived fibroblasts [49,55].

In vivo, injection-based delivery of in vitro-transcribed PE mRNA and canonical or synthetic pegRNA has been employed to introduce mutations in mouse zygotes [62] and to generate mouse models [89]. Furthermore, hydrodynamic tail vein injection of plasmid encoded prime editing components enabled pathogenic gene correction in the murine hepatocytes [69,90,91,92] and generation of a cancer model in vivo [43]. 

However, for treatment of inherited retinal disease, systemic delivery routes are not appropriate and strategies that can be applied via intravitreal or subretinal delivery are required.

### 4.1. Split-PE Delivery

For in vivo delivery of therapeutic gene editing cargo, viral vectors including adeno-associated virus (AAV), lentivirus, and adenovirus have widely been used and proven to be successful [93]. In the eye, AAV and lentiviral vectors are the most commonly used and of these, AAV vectors have the most convincing safety and efficacy profile [94,95] and are therefore the preferred choice for in vivo genome editing [93,96]. A gene therapy vector based on serotype AAV2 has already been approved by the US Food and Drug Administration (FDA) for delivery of a treatment of retinal degenerative disease [93]. However, the large size of the prime editor (>6.3kb), pegRNA, and nicking sgRNA provides a huge challenge these components exceed the packaging capacity of a single AAV vector (<4.7 kb) [97]. 

One strategy to bypass the AAV cargo limitation is the dual AAV approach. For this strategy, the transgene is split into an amino-terminal (N-PE) and a carboxy-terminal (C-PE) part which are packaged into separate AAV [96]. When both AAV vectors transduce the same cell, the full-length gene product may be reconstituted [98]. Reconstitution can be accomplished via DNA recombination due to overlap of identical elements in both vectors [99,100,101] and/or trans-splicing at pre-mRNA [102,103,104] RNA [105,106], or protein level [107]. Such dual vector strategies have been employed for large gene supplementation strategies with pre-clinical success in mouse models of retinal disease, indicating delivery of prime editing constructs by this route are a worthwhile pursuit [108,109,110,111]. Jang et al. [92] employed a *trans*-RNA-splicing dual AAV strategy, featuring two AAV8s encoding N-PE and C-PE, respectively, plus an additional AAV to deliver pegRNA and nicking sgRNA. Proof-of-principle delivery by intravitreal injection resulted in an average efficacy of 1.82% and 1.87%, for retina and RPE, respectively, with no observable off-targets or undesired indels. These results encouraged the authors to investigate the ability of prime editing to rescue the disease phenotype of the *rd12* mouse, a mouse model for LCA. The prime editing components were delivered subretinally with two tsAAV Vectors encoding the PE co-injected with an AAV Vector encoding pegRNA and mCherry. Six weeks post-injection, an average transduction efficacy of 23% and an editing efficacy of 6.4% in the RPE were observed, while no undesired edits or off-target effects were seen. Furthermore, ERG and optomotor response measurement showed functional rescue of the phenotype with improved visual function. Interestingly, compared to previous studies in the same animal model, prime editing outperformed the CRISPR nuclease-mediated HDR approach with higher editing efficacy and lower indel rates [112]. Furthermore, even though LV-mediated and AAV-mediated base editing yielded in higher editing efficacies (16% +/- 3% and 11% +/- 5%), relevant rates of bystander edits (17% and 7.7% +/- 5%) were seen within the same base editor window [113,114].

Delivery of PE components with the protein *trans*-splicing dual AAV strategy mediated by the self-sufficient polypeptide intein [107] has been attempted independently by several authors.

Utilizing a dual AAV8 protein-*trans*-splicing PE3 system based on Rma intein, Zhi et al. [34] observed editing efficacies of up to 42% in human HEK239T cells in vitro. However, when delivered to the murine retina via subretinal injection, a transversion mutation within *Dnmt1* could only be installed with a frequency of 1.71%. Interestingly, a similar approach published by the same authors featuring a split base editing dual AAV system in the murine retina, yielded editing efficacies as high as 25% [115]. The low efficacy of the trans-splicing dual AAV strategies may be due to the large size of the PE resulting in a limited choice of effective split-sites in the Cas9 protein and suboptimal vector design due to limited space for additional elements as a tissue specific promotor. Additionally, suboptimal splice-sites may lead to imperfect structure after re-assembly of the full-length PE or unwanted/truncated transgene constructs [43,91,96,101,116].

To address this issue, Zheng et al. developed a truncated PE, lacking the RNase H domain of RT, that could be utilized for precise editing both in vitro and in vivo, when delivered with the dual AAV split intein strategy [91]. The achieved editing efficacy was comparable with that of a full-length PE. Similarly, Gao et al. developed a codon- and size-optimized PE, with lacking RNase H domain and trimmed RT, named PE^co^-mini [116]. Dual-AAV delivery based on the Rma split-intein approach demonstrated 6% gene editing in the *Pcsk9* gene in the mouse liver. Likewise, utilizing a truncated PE with lacking RNase H domain and split intein-based dual AAV delivery, Bök et al. achieved editing efficacies of 15% in neonatal mice and 3.4% in adult mice livers [117]. These constructs have yet to be delivered to the retina but collectively, these efforts yield more compact prime editors that are applicable for dual split intein-based AAV-delivery in vivo and enable a more flexible vector design.

Others strategies to overcome the above mentioned issues are based on the assumption that the M-MLV-RT complex may be functioning in *trans* from another PE2 molecule not tethered to the target site [118] and the recent discovery that separated nSpCas9 and M-MMLV-RT proteins induced efficient prime editing [90,118]. Grünewald et al. developed the split-PE platform consisting of an engineered prime editor with an untethered, truncated M-MLV RT lacking the RNase H domain [118]. This platform showed similar editing efficacy and undesired indels rates compared to conventional PE2, respectively. Additionally, compared to the split-intein strategy, the split-PE platform showed superior prime editing outcomes in HEK239T cells, possibly due to the lacking step of reconstitution of the PE protein. Furthermore, the split-PE architecture enabled dual AAV delivery with one AAV encoding the nCas9 protein and the other encoding pegRNA, sgRNA, and RT. Similarly, Liu et al. described split prime editing (sPE), consisting of a nCas9 nickase that remains untethered from the M-MLV-RT [90]. The authors used this approach to facilitate genome editing in mammalian cells and in the mouse liver with editing efficacy and undesired indel comparable to the PE3 system. Furthermore, dual AAV- based delivery of sPE could facilitate correction of the disease-causing mutation in the mouse model of type I tyrosinemia, even though the editing efficacy was relatively low (1.3%). Lastly, this study showed that pegRNAs can successfully be split into a sgRNA and a circular RT-PBS (petRNA) with increased stability and flexibility.

Hence, these approaches address the size-related delivery issue for prime editing components and may therefore be an attractive option for in vivo delivery to the retina, but have not been tested for this purpose yet.

### 4.2. Single PE Construct Delivery 

Another strategy to meet the requirement for limited AAV cargo capacity may be utilization of alternative Cas9 variants and orthologues [119]. For prime editing, several research groups have investigated in SaCas9, SaCas9KKH, SauriCas9, and CjCas9 [43,55,117]. However, future work is required to improve their efficacy. 

Compared to AAV, adenoviral vectors have the disadvantage of higher immunogenicity, which may be a safety concern for clinical translation. However, they hold the advantage of a larger cargo capacity and can effectively transduce most tissue types [117,120]. Böck et al. systemically administered human adenoviral vectors 5 (AdV) encoding PE components into newborn mice and achieved editing efficacies of 58% and 11.1% for the *Dnmt1* locus and the *Pah(enu2)* locus, respectively [117]. Despite the high efficacy, this delivery approach has limitations in terms of clinical translation due the requirement of high vector doses and induction of immune response to vector and PE.

Wang et al. utilized a fully viral gene-deleted adenoviral vector (AdVP) with a packaging capacity of 30 kb, for an all-in-one transfer of full-length prime editing components [121]. This approach was tested in various human cells and resulted in efficacies up to 90%. However, adenoviral vectors are not commonly used in retinal gene therapy given the superior safety and performance profile of AAV vectors. 

Lentiviral vectors also offer larger cargo capacity and are being developed for treatment of retinal disease [122] but currently show reduced transduction profiles compared to AAV vectors with transduction typically localized and limited to the RPE [123]. Lentiviral vectors may therefore be considered for RPE targeting and indeed have been used to deliver full length ABE constructs in *rd12* mice, achieving up to 29% correction of the mutation in RPE cells with minimal undesired indels and off-target. Furthermore, treatment restored RPE65 expression and led to functional retinal and visual recovery [114]. 

Similarly, virus-like particles have been developed for full length base editing construct packaging and subretinal delivery to *rd12* mice yielded correction rates of up to 21% [124]. Despite these encouraging results, the correction of mutations that cause most inherited retinal diseases will require efficient transduction of the photoreceptor cells.

Delivery of full-length prime editing constructs may therefore need an alternative strategy involving direct delivery of the DNA constructs either in plasmid form or as minicircles, which have advantages over plasmid structures due to the removal of the bacterial elements [125]. Naked DNA can be delivered to the retina by electroporation and has been used to deliver CRISPR editing constructs to the *rd10* mouse model [126]. Non-viral delivery based on organic or inorganic nanoparticles may be another option to circumvent the size-related issue of the prime editing cargo [127]. Previous studies have shown delivery of gene therapeutic cargo to the murine retina in vivo using lipid nanoparticles (LNPs) [128], supramolecular nanoparticles [129], and carbon nanoparticles [130]. Recently, Herrera-Barrera et al., developed peptid-conjugated LNPs, that enable delivery of mRNA cargo to the neural retina in mice and nonhuman primates in vivo [131].

Prime editing is still a new genome editing tool, and therefore testing in vivo and particularly in the retina has, to date, been limited, but it is clear that several promising modes of delivery offer great potential. However, further optimization of the vehicles and test of safety are essential before moving forward to clinical application.

## 5. Conclusions

**Figure 4:**
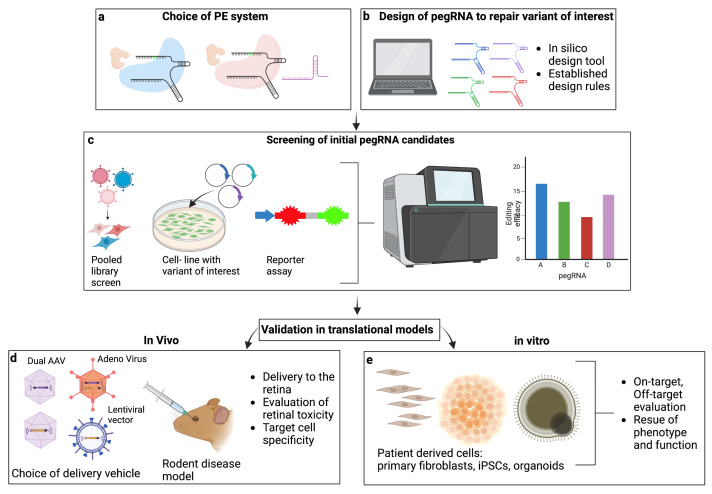
Workflow for the development and validation of prime editing as a treatment approach to target pathological variants causative for IRD. (**a**) A suitable prime editing system (i.e., PE2, PE3) and PE architecture (i.e., PEmax) are chosen. (**b**) pegRNAs to repair the variant of interest are designed using established designrules and in silico tools. (**c**) An initial screen of pegRNA candidates is performed using cell-lines containing the variant of interest or a reporter assay. Alternatively, a high throughput screen based on a pooled lentiviral library can be employed. Prime editing efficacy is evaluated by NGS or sanger sequencing. (**d**,**e**) The best performing pegRNAs are validated in translational models in vivo and in vitro. (**d**) Rodent disease models can be used to test different delivery vehicles for efficient delivery to the retina, to evaluate retinal toxicity, target cell specificity and for some models, rescue of phenotype and function. (**e**) Patient derived cells, i.e., Fibroblast, iPSC or organoids can be used for on-target evaluation and to evaluate rescue of phenotype and function.

Prime editing is a new and exciting technology that offers great potential to correct mutations that cause currently untreatable inherited retinal disease. Recently, Fry et al. conducted a cross-sectional study to identify the prevalence of single-nucleotide variant amendable to base editing in six recessively inherited genes which are associated with IRD and are too large for gene augmentation with a single AAV vector [132]. The authors found that 53% of the pathological variants in *ABCA4*, *CDH23*, *CEP290*, *EYS*, *MYO7A*, and *USH2A* could potentially be corrected via BE, leaving approximately 47% variants behind, including those caused by transversions, insertions, and deletions. Similarly, Kaukonen et al. [133] evaluated the potential of base editing based on SpCas9 and SaCas9, respectively, to correct pathogenic variants in the *rhodopsin (RHO)* gene causative for autosomal dominant RP (sdRP). The authors found that BE could theoretically correct 55% of all analyzed variants. However, this number was further restricted by PAM site availability and high incidence of likely bystander edits. The target scope of prime editing expands on this correction potential as it includes all 12 transversion mutation, insertions and deletions, and may therefore correct variants not amendable to base editing. Hence, prime editing may be a feasible approach to treat a large number of pathological variants causative for IRD. Figure 4 summarizes the essential steps to achieve efficient prime editing as discussed in this review and illustrates a potentional workflow to develop and evaluate prime editing based treatments for IRDs. 

Further studies are needed to refine prime editing efficacy in vivo, optimize retinal delivery, and address safety concerns including the amount of undesired editing byproducts and OFF-targets. 

## Figures and Tables

**Table 1:** Advances in the Prime editing technology.

Name	Mechanism/Improvement	Components	Editing Efficacy	Undesired Indels	Ref
PE1	Cas9 Nickase (H840A) + Wildtype M-MLV RT	PE1 + pegRNA	0.7–17%	0.2% +/- 0.1%	[35]
PE2	Modified M-MLV RT (D200N/LL603W/T330P/T306K/W313F)	PE2 + pegRNA	1.6–5.1 fold increase compared to PE1		[35]
PE3	Additional sgRNA nicking the non-edited strand -> favors incorporation of edited strand	PE2 + pegRNA+ nicking sgRNA	1.5–4.2 fold increase compared to PE2, up to 55%	Increased compared to PE2	[35]
PE3b	Nicking sgRNA with a spacer that matches the edited strand only	PE3 + pegRNA + nicking sgRNA with protospacer that only matches the edited strand	Similar to PE3	13-fold decrease compared to PE3	[35]
PE*	Enhanced nuclear localization of the Prime editor	PE3/PE2 (with optimized NLS) + pegRNA	1.3 to 3.4 fold increase compared to PE2	Same or slightly higher levels than conventional PE2 and PE3	[43]
hyPE2	Addition of a Rad51 DNA-bin binding protein domain to stabilize the ssDNA that is used as a primer for reverse transcription	PE2 (with Rad51 DNA-binding domain) + pegRNA	1.4 or 1.5 fold increase compared to PE2	Slightly decreased compared to PE2	[44]
IN-PE	Fusion of a dual peptide (IGFpm1-NFATC2IPp1) to PE 2 increases translation efficiency	IGFpm1-NFATC2IPp1 + pegRNA	Increased compared to PE2 (median in different cell-lines: 1.35X in A549, 1.92X in HCT-116, 1.17X in HEK293T, 1.78X in U2OS)		[46]
ePPE	Engineered M-MLV RT (removal of ribonuclease H domain + addition of viral nucleocapsid protein with chaperon activity)	PPE (with modified M-MLV RT) + pegRNA	5.8-fold increase compared to PPE	Similar to PPE	[47]
Prime editing with epegRNA	Stabilization of the 3´terminus of pegRNA with structured RNA motifs (evopreQ_1_ or mpknot)	PE3 + pegRNA (with evopreQ_1_/mpknot)	3–4 fold increase compared to PE3	Similar to PE3	[49]
xrPE	Stabilization of 3´terminus of pegRNA with viral exoribonuclease-resistant RNA motif (xRNA)	PE3 + pegRNA (with xrRNA)	2.5–4.5 fold increase compared to PE3	Similar to PE3	[50]
G-PE	Stabilization of 3´terminus of pegRNA with a structured RNA motif (human telomerase RNA (hTR) G-quadruplex modification)	PE + pegRNA (with hTR G-quadruplex formation)	Similar to xrPE and prime editing with epegRNA, up to 1.9 fold increase compared to conventional prime editing	Higher indel frequency than conventional prime editing	[51]
sPE	Introduction of same-sense mutations in RTT	PE3/PE5 + pegRNA (with SSM in RTT)	353-fold increase (for base conversions) compared to PE3	Similar to PE3	[52]
aPE	Stabilization of pegRNA secondary structure	PE3/PE5 + pegRNA (modified)	2.77-fold increase (for indel-editing) compared to PE3	Similar to PE3	[52]
Enhanced PE (ePE)	Prevention of pegRNA circulation by a Csy4 recognition site	PE3 (Csy4-T2A) + pegRNA (with Cys recognition site)	1.9 fold increased compared to PE3	Increased compared to PE3	[53]
PE4	Co-delivery of engineered MMR-inhibiting protein (MLH1d)	PE2 + co-delivered MLH1dn + pegRNA	7-fold increase compared to PE2	3.4-fold improvement of edit:indel ratio in MMR proficient cells	[55]
PE5	Co-delivery of engineered MMR-inhibiting protein (MLH1d)	PE3 + co-delivered MLH1dn + pegRNA	2.0-fold increase compared to PE3	3.4-fold improvement of edit:indel ratio in MMR proficient cells	[55]
PE4 MAX	Co-delivery of engineered MMR-inhibiting protein (MLH1d)+ optimized PE architecture (human codan optimized RT, c-Myc NLS, linker, R221K, N394K mutation of SpCas))	PE2 (optimized) + co-delivered MLH1dn + pegRNA /epegRNA	2.8 fold improvement on average in HeLa cells compared to PE2 architecture	Decreased compared to PE2	[55]
PE5 MAX	Co-delivery of engineered MMR-inhibiting protein (MLH1d)+ optimized PE architecture (human codan optimized RT, c-Myc NLS, linker, R221K, N394K mutation of SpCas))	PE3 (optimized) + co-delivered MLH1dn + pegRNA (or epegRNA) + nicking sgRNA	2.8 fold improvement on average in HeLa cells compared to PE2 architecture	1.2 fold decrease compared to PE5	[55]
Enhanced PE	Opening chromatin structure at target site using proximal dead sgRNA (dsgRNA) and chromatin-modulating peptides	PE3 + pegRNA + dsgRNA + CMP	2.55–5.11-fold increase compared to PE3		[62]
Prime editing with HDACi	Opening chromatin structure at target site using HDAC inhibitors	PE3 + pegRNA + HDAC inhibitor	Compared to PE3: Increased for insertions (1.37-fold) and deletions (1.40-fold), decreased for installation of point mutations.		[63]
twinPE	Two pegRNAs, each targeting the opposite strand and encoding strands complementary to each other	PE2 + 2 pegRNAs	For 108-bp insertion: 20-fold increase compared to PE3	Fewer indels compared to paired Cas9 nuclease strategy	[64]
GRAND	Two pegRNAs, each targeting the opposite strand and encoding strands complementary to each other	PE2 + 2 pegRNAs	up to 63% for 150 bp insertion		[65]
HOPE	Two pegRNAs, each targeting the opposite strand and encoding strands complementary to each other	PE2 + 2 pegRNAs	higher editing efficacy compared to PE2/PE3	Fewer indels compared to PE3	[66]
PRIME-Del	Two pegRNAs, targeting the opposite strand, encoding for strands complementary to each other and the DNA sequence upstream of the nick on opposite strand	PE2 + 2 pegRNAs	1–30% editing efficacy	Fewer undesired indels compared to paired Cas9 nuclease strategy	[67]
Bi-PE	Two pegRNAs, targeting the opposite strand, encoding for strands complementary to each other and the DNA sequence upstream of the nick on opposite strand	PE2 + 2 pegRNAs	Compared to PE3: increased for conversions of multiple bases, no improved for single base conversions	[68]	[68]
PEDAR	Two pegRNAs, targeting opposite strands, Cas9 nuclease	PE-Cas9 (nuclease) + pegRNA	similar to PRIME-Del	Increased compared to PRIME-del	[69]
PASTE	CRISPR-Cas9 nickase fused to a reverse transcriptase and an additional serine integrase	Cas9nickase+ RT + serine integrase	5–50% efficacy for different inserts		[70]
